# Why diabetes-related foot ulcers fail to heal despite successful revascularization: closing the patency-to-healing gap: a narrative review

**DOI:** 10.12701/jyms.2026.43.60

**Published:** 2026-09-02

**Authors:** Mohamed Magdy Mohamed Badr

**Affiliations:** 1 Wound Care and Diabetic Foot Unit, Armed Forces Rehabilitation Center, Giza, Egypt; 2 Wound OnCall Center, Cairo, Egypt

**Keywords:** Chronic limb-threatening ischemia, Diabetic Foot, Microcirculation, Peripheral arterial disease, Vascular surgical procedures, Wound healing

## Abstract

Revascularization is central to limb salvage when a diabetes-related foot ulcer is complicated by peripheral artery disease. However, an open artery does not guarantee wound healing. This patency-to-healing gap arises because repair requires regional perfusion, infection control, mechanical protection, viable tissues, physiological reserves, and coordinated care. This focused narrative review searched PubMed/MEDLINE through June 30, 2026, and supplemented the search with guidelines, landmark trials, and citation chaining. The BEST-CLI and BASIL-2 studies showed that the results depended on the population, anatomy, and endpoints studied. Neither technical success nor composite limb endpoint confirmed adequate wound-territory perfusion. This review separates technical, hemodynamic, regional tissue, and clinical success and offers a time-linked approach to reassessment. A decrease of approximately 50% in the wound area by 4 weeks may signal the need to revisit the plan; however, the threshold was derived largely from nonischemic neuropathic ulcers and has not been validated as a decision rule after revascularization. It is particularly unreliable after major debridement, in dry gangrene, or when the baseline is unstable. PIPPP (perfusion, infection, pressure, patient, and plan) is presented as an unvalidated, hypothesis-generating checklist that overlaps with, rather than replaces, the International Working Group on the Diabetic Foot; wound, ischemia, and foot infection; Global Limb Anatomic Staging System; and Global Vascular Guideline structures. Prospective validation is required before clinical effectiveness can be achieved. Durable healing requires more than a patent vessel; tissue perfusion must be confirmed, recurrent ischemia monitored, infection controlled, pressure relieved, systemic problems addressed, and responsibility for follow-up made clear.

## Introduction

Diabetes-related foot ulceration carries heavy human costs due to infection, repeated admissions, limb loss, and early death [1]. When peripheral artery disease (PAD) is a part of the picture, healing becomes less likely, and limb preservation is more difficult. Revascularization is essential when ischemia contributes to tissue loss. Current intersocietal guidelines recommend complete arterial imaging, restoration of inline flow to at least one foot artery when feasible, and objective confirmation that perfusion has improved [2]. The Global Vascular Guidelines (GVG) place the procedure within a wider range of clinical decisions: patient risk, limb severity, and anatomic complexity. The Global Limb Anatomic Staging System (GLASS) then describes the target artery path and complexity of the disease [3].

The word “successful” should be considered carefully, as the BEST-CLI and BASIL-2 studies make clear. In BEST-CLI, bypass with an adequate great saphenous vein produced fewer major adverse limb events or deaths than an endovascular-first strategy in the suitable-vein cohort, and the outcomes did not differ significantly when an adequate vein was unavailable [4]. BASIL-2 studied patients who required infrapopliteal revascularization and found better amputation-free survival with the best-endovascular-treatment-first strategy than with the vein-bypass-first strategy, largely because of the difference in mortality [5]. At first glance, these findings may appear contradictory. They are not directly comparable because the trials enrolled different patients, examined different strategies, and used composite outcomes with different clinical drivers. Neither trial establishes a universal procedural hierarchy nor allows us to equate a patent vessel with a healed ulcer.

The underlying biology discussed herein is well-established; however, the primary contribution of this review is its practicality. The review first separates technical, hemodynamic, regional tissue, and clinical success so that open reconstruction is not mistaken for a perfused wound. The review then transforms familiar guideline domains into a time-linked sequence for a wound that stalls after revascularization. Finally, it sets clear limits on the 4-week wound-area trajectory when the rule is borrowed from neuropathic ulcers and applied to ischemic wounds. Perfusion, infection, pressure, patient, and plan (PIPPP) is offered only as a bedside memory aid, layered onto International Working Group on the Diabetic Foot (IWGDF); wound, ischemia, and foot infection (WIfI); GLASS; and GVG structures. PIPPP is not a new score and should not be treated as one.

## Search methods and scope

This focused narrative review was prepared with reference to the Scale for the Assessment of Narrative Review Articles (SANRA) domains for quality of narrative reviews [6]. PubMed/MEDLINE was searched from January 1, 2000, through June 30, 2026. The reproducible core search was performed as follows: ((“diabetic foot”[Title/Abstract] OR “diabetes-related foot ulcer”[Title/Abstract] OR “diabetic foot ulcer”[Title/Abstract]) AND (revascularization[Title/Abstract] OR revascularisation[Title/Abstract] OR “chronic limb-threatening ischemia”[Title/Abstract] OR “peripheral artery disease”[Title/Abstract]) AND (“wound healing”[Title/Abstract] OR healing[Title/Abstract] OR perfusion[Title/Abstract] OR microcirculation[Title/Abstract] OR offloading[Title/Abstract] OR infection[Title/Abstract] OR osteomyelitis[Title/Abstract])).

In total, 596 records were identified from the query. Because the purpose was focused on synthesis rather than exhaustive effect estimation, selection was relevance-based rather than a PRISMA (Preferred Reporting Items for Systematic Reviews and Meta-Analyses) process. Twenty-two database records were retained after single-author title/abstract assessment and full-text review. Eight additional landmark trials, guidelines, and other clinically relevant sources were identified through targeted searches and backward citation chaining, yielding 30 clinical evidence sources in the final synthesis. The SANRA publication [6] was used solely as a methodological framework and was not counted among these 30 sources. The eligible sources were English-language adult human studies, systematic reviews, trials, and multidisciplinary guidelines addressing diabetes-related foot ulcer (DRFU) or chronic limb-threatening ischemia (CLTI), revascularization outcomes, tissue perfusion, infection, offloading, systemic prognostic factors, and care delivery. At full-text review, records were excluded when they did not provide a clinically relevant limb or healing endpoint, addressed non-foot or non-diabetic wounds without separable data, duplicated evidence already represented by a higher-level source, or lacked direct relevance to post-revascularization healing. Animal or in vitro studies, case reports, and isolated device reports were also excluded. No meta-analysis was performed because ulcer definitions, procedures, perfusion tests, follow-up intervals, and healing endpoints were heterogeneous.

## Revascularization success is multidimensional

The term “successful revascularization” can conceal four different outcomes (Table 1). The target lesion may have been treated, and the bypass may have been opened (i.e., technical success). Limb blood flow may have measurably improved (i.e., hemodynamic success). Oxygenated blood may have reached the ulcer bed and margin (i.e., regional tissue success). Only then could the outcomes that matter most to the patient (i.e., progressive repair, durable closure, preservation of the limb, and useful function) be assessed. The four levels are connected but not interchangeable. The relationship between macrovascular patency, tissue-level perfusion, and durable healing is illustrated in Fig. 1.

The BEST-CLI and BASIL-2 studies also showed that a trial’s primary composite endpoint is not a direct assay of wound biology (Table 2) [4,5]. Major adverse limb events, reintervention, major amputation, and death matter greatly; however, the same composite result may contain different proportions of mortality, reintervention, and limb loss. The definitions and ascertainments of wound healing are not uniform. In practice, the operator’s completion image, the vascular laboratory results, and the longitudinal measurements made by the wound clinician answer different questions.

The ankle-brachial index can be misleading in patients with diabetes because medial calcification may cause incompressibility. Toe pressure may be impossible after toe loss, or a territory different from the ulcer may be assessed. Transcutaneous oxygen pressure (TcPO_2_) and skin perfusion pressure (SPP) provide more tissue-oriented information but are affected by edema, temperature, inflammation, probe location, and technique [2,7]. A toe pressure of ≥30 mmHg, TcPO_2_ of ≥25 mmHg, or SPP of ≥40 mmHg increases the probability of, but does not guarantee healing [2,7]. A recent study comparing noninvasive tests after endovascular treatment further supports the interpretation of perfusion measurements in anatomical and clinical contexts rather than using a single universal cutoff [8].

WIfI integrates wound extent, ischemia, and foot infection and therefore complements anatomy-focused staging [9]. After revascularization, repeating the ischemia component (and, when clinically useful, restaging the complete WIfI profile) makes the reassessment explicit: Did ischemia improve, and is the remaining risk now driven by the wound or infection? Evidence that formal post-procedural restaging itself improves outcomes is limited; therefore, it should be viewed as a structured reassessment rather than as a proven intervention.

## Vascular causes of persistent nonhealing

### 1. Residual disease, early failure, and restenosis

A technically satisfactory procedure can leave significant inflow, tibial, pedal, or collateral disease. A strong initial result may be eroded by recoil, dissection, thrombosis, distal embolization, conduit failure, competitive flow, and early restenosis. The completion angiogram shows what is true at that moment. It does not promise durable perfusion.

When a wound stalls, the most useful comparison is with the immediate post-procedural baseline, not with memory or a vague note that the pulse was “better.” Clinicians should compare pulses and Doppler waveforms and then use duplex ultrasound to assess a graft or accessible treated segment. Toe pressure, TcPO_2_, and SPP can indicate whether tissue perfusion has improved, and cross-sectional or catheter angiography may be needed when recurrent ischemia is plausible [2,3]. New necrosis, pain, coolness, poorer waveforms, and the loss of previously documented perfusion gain should lower the threshold for reassessment.

### 2. Global Limb Anatomic Staging System, target artery path, and pedal circulation

GLASS describes the complexity of the preferred target artery path and supports patient-, limb-, and anatomy-based revascularization strategies [3]. However, for tissue-loss presentations, restoring flow to the ankle does not necessarily establish a functional circuit through the pedal arch and into the wound. Severe inframalleolar disease is associated with adverse limb outcomes after endovascular treatment of CLTI [10]. A patent anterior tibial artery may deliver limited nutritive flow to a plantar or heel wound if the dorsalis pedis–plantar connections are poor.

Therefore, a review of the original angiogram should determine whether the wound territory is opacified, whether the pedal arch is filled, whether collateral flow is robust, and whether additional distal reconstruction is feasible. This is not an argument that every patient requires pedal intervention, but rather that “one-vessel runoff” is an anatomical description, not proof of regional tissue success.

### 3. Angiosome mismatch

Direct revascularization of the artery supplying the angiosome of the ulcer may improve healing in selected patients, particularly those with poor collateral networks. However, the evidence is heterogeneous, observational confounding is substantial, and direct targeting may be impossible or less consequential when the pedal arch redistributes the flow [2,11]. However, the conclusion should remain modest. Regional perfusion matters, but the angiosome concept should inform and not dictate a strategy.

### 4. Macrovascular patency without proven microcirculatory recovery

Nutrient delivery depends on arterioles, capillary recruitment, endothelial function, erythrocyte transit, oxygen diffusion, and venous and lymphatic clearance. Diabetes impairs several of these processes. Macrovascular repair can increase pressure without normalizing the wound microenvironment. Transient post-revascularization dysfunction may reflect impaired autoregulation, endothelial reactivity, oxidative and inflammatory injuries, capillary no-reflow phenomenon from leukocytes, endothelial swelling, and interstitial edema. These processes can delay nutritive oxygen delivery despite the restored conduit flow [12].

A small 2005 cohort study observed that TcPO_2_ may improve gradually over several weeks after a technically successful angioplasty [13]. That study offers useful physiological evidence but is too small and dated to prescribe a universal surveillance time. A 2024 cohort study using serial TcPO_2_ measurements found postoperative changes and prognostic associations, supporting repeated rather than one-off assessments, but it also did not establish an optimal schedule [14]. Similarly, laser-speckle studies have associated ulcer-region microcirculation with healing [15]. The assumption that microcirculation takes weeks to improve should not delay intervention for a deteriorating wound. Thus, an early baseline is useful. The decision of when to repeat intervention should depend on the behavior of the wound and the patient’s risk; any clinical decline deserves prompt investigation.

### 5. Edema and impaired outflow

Interstitial edema increases the diffusion distance, increases tissue pressure, promotes maceration, and may blunt the oxygen benefits gained from revascularization. The causes include heart or renal failure, dependency, infection, venous hypertension, lymphatic dysfunction, and postoperative inflammation. Compression may help select patients, but arterial safety must be considered and the compression reassessed after revascularization [16]. Persistent swelling is, therefore, a possible mechanism of nonhealing and not a cosmetic finding.

### 6. No-option chronic limb-threatening ischemia and deep venous arterialization

Some patients have no conventional surgical or endovascular target. In the PROMISE II trial, transcatheter deep venous arterialization was technically successful in 99% of 105 selected no-option patients; the 6-month amputation-free survival rate was 66.1%, while complete and ongoing wound healing was documented in 25% and 51% of patients who were evaluable, respectively [17]. These single-arm results are encouraging but cannot be equated with comparative evidence for conventional revascularization. Deep venous arterialization should be discussed in expert multidisciplinary centers for carefully selected patients with no-option CLTI, with explicit acknowledgment of procedural burden, reintervention, uncertain generalizability, and the continued need for wound care.

## Local wound factors that can defeat restored flow

### 1. Infection and source control

Revascularization helps immune cells and antibiotics reach the foot. It does not drain pus, remove dead tissue, or excise the infected bone. Persistent erythema, new pain, malodor, friable tissue, purulence, increased exudates, systemic deterioration, or unexplained hyperglycemia should trigger immediate reassessment. The IWGDF/Infectious Diseases Society of America guidelines recommend clinical diagnosis, appropriate deep-tissue sampling when a culture is needed, urgent drainage of deep collections, and structured evaluation of suspected osteomyelitis [18]. No antibiotic regimen can target retained necrosis or undrained compartments.

### 2. Osteomyelitis and residual infected bone

Osteomyelitis may sustain inflammation and drainage after soft-tissue perfusion improves. Management can be medical, surgical, or combined, depending on the anatomy, perfusion, destruction, soft-tissue coverage, preference, and operative risk. A previous meta-analysis reported heterogeneous success rates and could not establish the universal superiority of one strategy [19]. The practical point is diagnostic: when a revascularized ulcer remains deep, probes to the bone, drains persistently, or repeatedly breaks down, residual bone infection must be actively reconsidered.

### 3. Devitalized tissue and wound-bed failure

Restored flow cannot convert necrotic tissues into viable tissues. Debridement should be individualized according to perfusion, tissue viability, anatomy, pain, and bleeding risk. Aggressive excision before adequate flow can enlarge the ischemic injury, and failure to remove nonviable tissue after adequate flow can perpetuate inflammation and infection. The wound should be assessed for granulation, edge advancement, undermining, tunneling, exposed tendon or bone, dead space, and closure tension.

IWGDF 2023 makes conditional recommendations for selected adjuncts, including sucrose octasulfate dressings, placental-derived products, and topical oxygen, in defined circumstances [20]. These options should not be disregarded. They are best considered after adequate standard care has been documented and residual ischemia, infection, and mechanical loading have been addressed. “Adjunct, not substitute” is a sequencing principle, not a judgment that adjuncts lack value.

### 4. Mechanical load, shear, and actual offloading exposure

Blood flow cannot heal tissues that are repeatedly traumatized. Neuropathy eliminates pain as a protective warning, whereas deformity, limited joint mobility, Charcot architecture, bony prominence, and poorly fitting footwear focus the pressure and shear. When not contraindicated, nonremovable knee-high devices are generally the first choice for uncomplicated neuropathic plantar forefoot or midfoot ulcers [21]. Randomized studies have shown better healing with total-contact casting or irremovable walkers than with removable alternatives [22,23], and activity monitoring demonstrates that removable devices may be worn for only a minority of daily steps [24].

A prescribed offloading device is not necessarily an offloading device worn throughout the day. It is essential to inspect the device and its wear pattern, observe the patient’s walk and transfer, and determine what occurs at home rather than accepting a simple affirmation of adherence. The appropriate device may also change after revascularization as edema, infection, balance, or wound location evolves.

### 5. Defects that require reconstruction

Large defects, exposed tendon or bone, loss of plantar padding, dead space, unstable amputation flaps, and excessive closure tension can exceed local regenerative capacity, even when perfusion is adequate. Staged debridement, negative pressure wound therapy, tendon balancing, bone resection, grafting, local or free tissue transfer, and delayed closure may also be required. Rather than closure at any cost, the goal is to establish a durable, plantigrade, shoeable, and pressure-tolerant foot.

## Patient-level barriers

### 1. Kidney disease

Chronic kidney disease clusters with medial calcification, distal PAD, anemia, immune dysfunction, edema, protein-energy wasting, and high cardiovascular risk. A 2021 systematic review and meta-analysis found worse limb and survival outcomes after lower extremity arterial intervention in patients with chronic or end-stage kidney disease [25]. Similarly, contemporary BEST-CLI subgroup data showed that increased renal disease severity was associated with worse revascularization outcomes [26]. A 1995 surgical series described the failure of foot salvage despite graft patency in end-stage renal disease [27]; this is used only as a historical illustration, not as contemporary causal proof or a basis for modality selection. Modern evidence supports a softer but important conclusion: in renal disease, patent reconstruction may coexist with poor healing, because systemic and tissue-level risks remain high.

### 2. Anemia and oxygen delivery

Anemia reduces arterial oxygen content and can limit the benefits of restored flow. The MEDFUN study associated anemia with adverse outcomes in a single-country observational cohort [28], and a separate 654-patient retrospective cohort study associated low hemoglobin levels with major amputation and mortality [29]. These studies support risk recognition, not causality or a specific transfusion target. Clinicians should evaluate bleeding, iron deficiency, inflammation, kidney disease, hemolysis, and nutritional deficiency; however, whether correcting anemia accelerates DRFU closure remains uncertain.

### 3. Glycemia, nutrition, smoking, and physiological reserve

Hyperglycemia impairs host defense and repair, and aggressive treatment can cause hypoglycemia in patients who are frail or renally impaired. The aim is to ensure safe metabolic stability. Nutritional assessment should include weight trajectory, appetite, protein and energy intake, food access, renal restrictions, and plausible deficiencies; observational associations are stronger than evidence for indiscriminate supplementation [30]. Smoking, heart failure, hypotension, arrhythmias, dehydration, and interruption of antithrombotic or lipid-lowering therapies can also reduce effective tissue delivery or threaten reconstruction [2,3].

## Measuring response without overextending the 4-week rule

### 1. Establish a reproducible baseline

After revascularization and any major debridement, the length, width, area, depth, tissue composition, drainage, undermining, exposed structures, periwound condition, infection status, and an image with a scale should be documented. The same method should be used at follow-up. The percentage area reduction is calculated as follows:

Percentage area reduction=[(baseline area−current area)/baseline area]×100.

Area alone can be misleading. A wound may become narrower if a deep infection persists. It may enlarge after appropriate debridement, even as the tissue becomes healthier. Depth, viable granulation, edge advancement, necrosis, drainage, pain, and perfusion indicate the remainder of the story.

### 2. The approximately 50% threshold at 4 weeks is an extrapolation

Sheehan et al. [31] found that a 4-week percentage area reduction predicted 12-week healing in a post hoc analysis of a becaplermin trial population. That cohort predominantly presented with neuropathic ulcers, and patients with clinically meaningful ischemia were excluded. Therefore, a threshold was not derived or validated as an escalation rule for post-revascularization ischemic wounds, which is the population addressed in this review.

This limitation changes the role of the number. An area reduction of approximately 50% at 4 weeks can prompt the team to stop and review the entire plan. It should not decide by itself whether treatment has passed or failed, that care must be stopped, or that the vascular procedure has failed. The calculation becomes especially fragile when the baseline is recorded before major debridement, dry gangrene awaits demarcation, infection control changes the shape of the wound, or edema and measurement techniques are unstable. A deteriorating wound cannot wait 4 weeks. Therefore, a stable but slow wound needs to be assessed. If a signal is used, the starting point should be clinically meaningful and should follow the documentation of perfusion, debridement status, infection control, and offloading.

## Perfusion, infection, pressure, patient, and plan: a proposed cognitive-forcing checklist

PIPPP is proposed as a memory aid when a wound does not follow the expected course (Table 3, Fig. 2). It is not a prognostic model, severity score, or validated clinical instrument. There are no data on inter-rater reliability, discrimination, calibration, feasibility, or outcome impact. Its first four domains intentionally overlap with the IWGDF and WIfI constructs. The fifth, Plan, is a care-process domain included because correct clinical decisions fail when ownership, timing, access, and escalation are unclear and not simply to complete the acronym.

At the bedside, this sequence can be used to avoid settling too early for a single explanation. A patent duplex ultrasound finding should not end the perfusion review if oxygenation at the wound edge did not improve. A positive swab should not distract from an undrained collection. The use of a prescribed walker should not be presumed. PIPPP generates questions for testing and does not provide answers. Prospective validation is needed before anyone can claim that its use improves outcomes.

## A time-linked post-revascularization pathway

The first reassessment should document the new vascular baseline, including pulses or Doppler waveforms, an objective perfusion measure when feasible, wound status, and procedural complications. From 1 to 2 weeks after revascularization, priorities include surveillance for ischemic deterioration, infection source control, debridement when safe, edema management, and a workable offloading strategy. From week 2 to 4, serial measurements should reveal whether the tissue quality, area, depth, and drainage are improving.

Post-revascularization WIfI reassessment can separate residual ischemia from persistent wound and infection burden, while a review of GLASS and the target artery path can clarify whether the original anatomical goal has been achieved [3,9]. Wounds that deteriorate at any time require urgent evaluation. A clinically stable wound that shows limited progress by approximately 4 weeks should trigger a complete PIPPP review, with the caveats above, and a new quantitative endpoint rather than the continuation of an unchanged plan (Table 4).

Multidisciplinary care refers to several specialties seeing the same patient on different days. The team needs one shared wound description, an agreed-upon vascular baseline, a named person responsible for each decision, rapid access to reimaging or surgery, and a date on which a lack of progress will change the plan. Without this connection, patients may become busy with appointments and receive fragmented care. Thus, the patency-to-healing gap is easier to observe and close.

## Evidence gaps

Research should separately report technical, hemodynamic, regional perfusion, wound-healing, limb, functional, and patient-reported outcomes. Trials require standardized definitions of baseline wound area, major debridement, complete epithelialization, recurrence, and time to heal. Serial tissue-perfusion studies should be performed to determine the best timing and meaningful changes after different revascularization strategies. Post-procedural WIfI restaging and pedal-arch or wound-territory perfusion require prospective evaluation. PIPPP should undergo content validation, usability testing, inter-rater reliability assessment, and comparative implementation studies before claims of clinical benefits are made.

## Limitations of this review

This was a single-author focused narrative review, not a systematic review. Screening and selection were not duplicated and there was no independent adjudication. Inclusion was purposive and may have been selective, despite a reproducible core search and citation chaining. No formal risk of bias or certainty of evidence assessment was performed and English language restrictions may have excluded relevant studies. The evidence base combines guidelines, randomized trials, systematic reviews, and observational cohorts with heterogeneous populations and endpoints; therefore, some mechanistic and prognostic statements remain indirect. These features make the synthesis susceptible to confirmation of the author’s central thesis. The review and PIPPP checklist should be read as a clinically reasoned framework that requires testing, and not as validated guidance.

## Conclusion

Revascularization is indispensable for many ischemic DRFUs but is still only one link in the chain. The wound must receive sufficient oxygenated blood, infection must be controlled, pressure must be relieved, the tissue must be capable of reconstruction, and the patient and care system must be able to sustain the plan. The BEST-CLI and BASIL-2 studies have sharpened this message; the meaning of success changes with the population, anatomy, comparator, and endpoint. The 50%-at-4-weeks trajectory may prompt reassessment; however, this is not a validated rule after revascularization. PIPPP is an untested checklist and is not a new score. Prospective validation is required before clinical effectiveness can be claimed. The practical task is straightforward to state, even when it is difficult to deliver, specifically to confirm what the procedure has achieved at the wound, find what still stands in the way of repair, and agree in advance when a lack of progress changes the plan.

## Figures and Tables

**Fig. 1.**
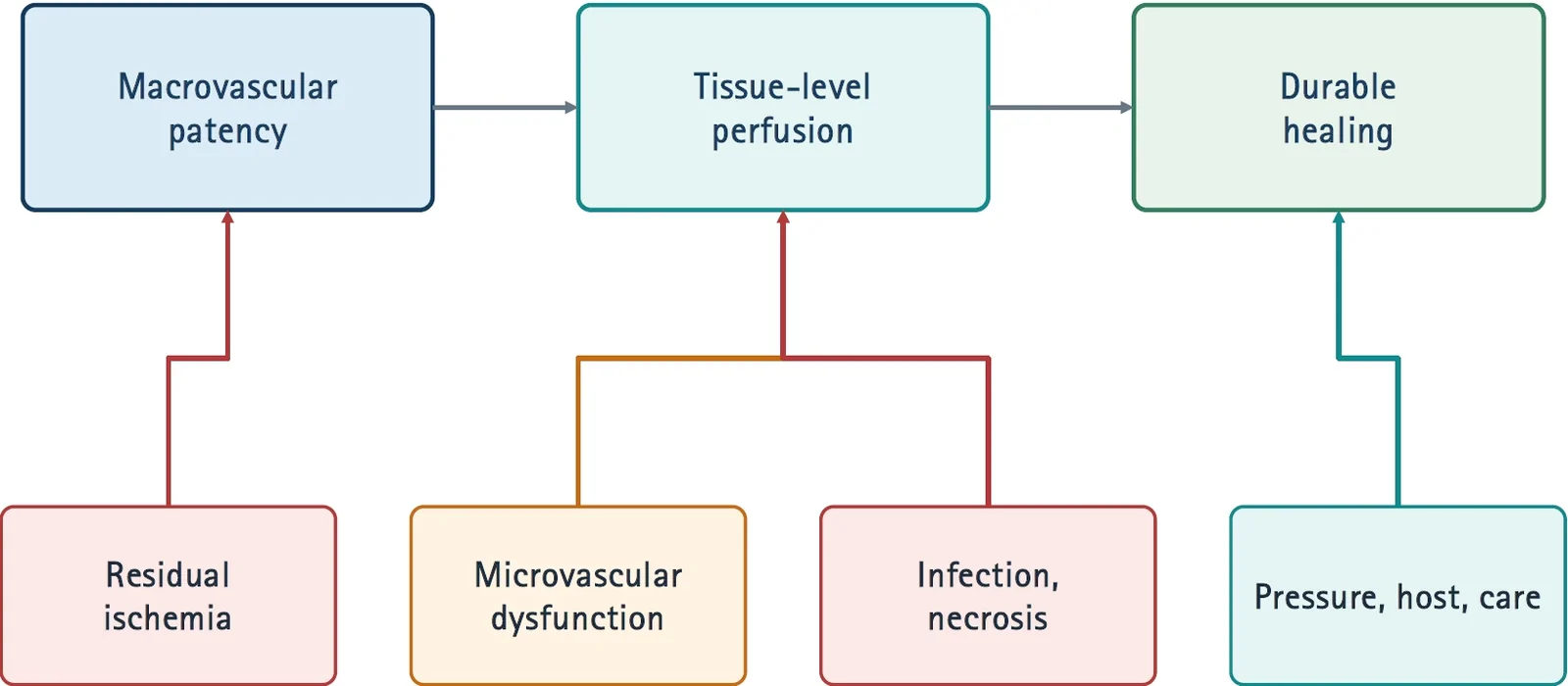
The patency-to-healing gap. Technical revascularization restores a macroscopic conduit, but complete healing requires adequate regional tissue perfusion plus control of infection, pressure, wound-bed pathology, systemic deficits, and care-process barriers. Failure at any downstream layer can prevent closure despite vessel patency.

**Fig. 2.**
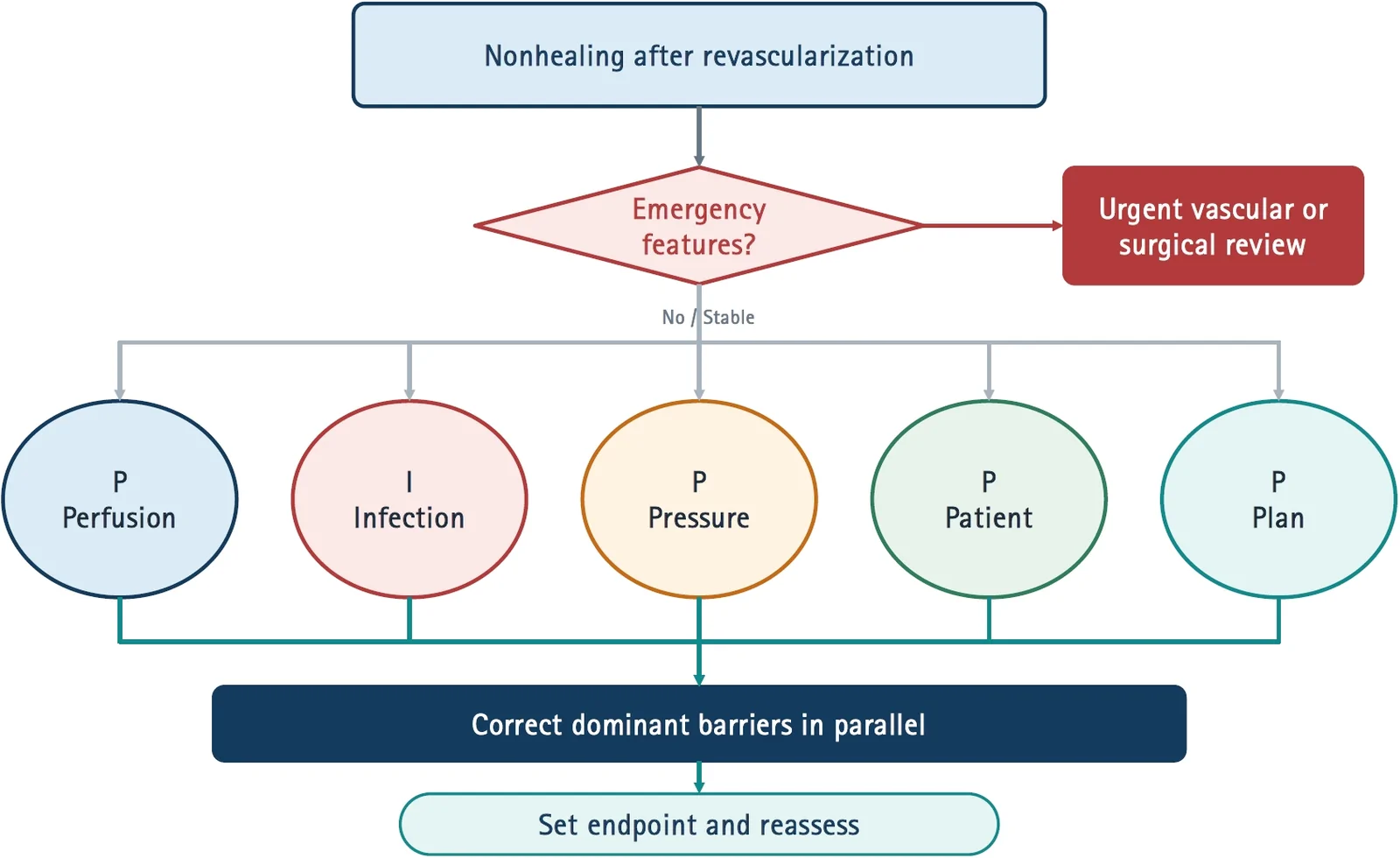
Proposed perfusion, infection, pressure, patient, and plan (PIPPP) reassessment algorithm. PIPPP is an unvalidated, hypothesis-generating cognitive-forcing checklist for a diabetes-related foot ulcer that deteriorates or follows an inadequate trajectory after revascularization. The approximately 50%-at-4-weeks signal is an extrapolated review prompt, not a validated decision rule in ischemic post-revascularization wounds. Prospective validation is required before clinical effectiveness can be claimed.

**Table 1.** Four levels of revascularization success

Level	Operational meaning	Useful evidence	Common false reassurance
Technical	Target lesion treated or bypass patent	Completion angiography; operative assessment; early duplex	“The artery is open, therefore the wound will heal”
Hemodynamic	Limb blood flow measurably improved	Waveforms; toe pressure; TcPO_2_; SPP; duplex velocities	A single improved value treated as definitive
Regional tissue	Oxygenated flow reaches the ulcer territory	Wound-adjacent TcPO_2_/SPP; pedal-arch filling; tissue appearance; serial trend	One-vessel runoff assumed to perfuse every wound
Clinical	Progressive repair, durable closure, limb and function preserved	Standardized serial measurement; epithelialization; recurrence-free follow-up; function	Patency or limb salvage substituted for wound healing

TcPO_2_, transcutaneous oxygen pressure; SPP, skin perfusion pressure.

**Table 2.** What the landmark CLTI trials clarify—and what they do not

Trial	Population and comparison	Principal message relevant to this review	What remains unanswered at wound level
BEST-CLI [4]	CLTI; bypass-first vs. endovascular-first; cohorts defined by adequate great saphenous vein	Strategy effect differed by conduit availability; reintervention and major limb events matter	Completion patency or the composite endpoint does not prove adequate wound-territory perfusion or closure
BASIL-2 [5]	CLTI requiring infrapopliteal treatment; vein-bypass-first vs. best-endovascular-treatment-first	Endovascular-first improved amputation-free survival, largely through a mortality difference	Composite survival can diverge from wound biology; healing definitions and regional perfusion remain separate outcomes

CLTI, chronic limb-threatening ischemia.

**Table 3.** Proposed perfusion, infection, pressure, patient, and plan (PIPPP): proposed, unvalidated reassessment prompts

Domain	Question	Possible action
Perfusion	Was tissue-level improvement documented? Are waveforms or pressures worse? Did the wound territory and pedal arch fill?	Repeat objective perfusion test; duplex; review angiography/GLASS target path; consider re-imaging or reintervention
Infection	Is there an undrained collection, necrosis, or osteomyelitis? Was sampling adequate?	Surgical review; deep-tissue or bone sampling; imaging; drainage/debridement; targeted antimicrobial therapy
Pressure	Is the wound still exposed to pressure or shear? Is the device worn and safe?	Inspect device and wear pattern; observe gait/transfers; modify or make nonremovable when appropriate; address deformity
Patient	Are renal disease, anemia, edema, nutrition, glycemia, smoking, or cardiac instability limiting repair?	Targeted medical and nutritional assessment; medication reconciliation; shared goals
Plan	Who owns each action? What is the next endpoint and date? Are access or adherence barriers unresolved?	Name responsible clinicians; set measurable target; arrange rapid escalation; align plan with patient priorities

GLASS, Global Limb Anatomic Staging System.

**Table 4.** Time-linked pathway after revascularization

Time point	Minimum reassessment	Escalation signal
24–72 hours or first review	Document vascular baseline, wound status, pain, temperature, waveforms, and objective perfusion when feasible	Acute ischemia, new necrosis, procedural complication, sepsis, deep collection
Week 1–2	Reassess perfusion and infection; debride when safe; establish offloading; measure and photograph wound	New tissue loss, spreading infection, worsening waveform/perfusion, device injury
Week 2–4	Trend area, depth, tissue quality, drainage; verify actual offloading; address edema and systemic deficits	Static/enlarging wound, poor granulation, persistent drainage, exposed bone, loss of perfusion gain
Approximately week 4	Apply contextual trajectory assessment; complete PIPPP review if progress is inadequate	<50% area reduction as a review prompt—not a validated rule—or any clinically important deterioration
After closure	Protective footwear, callus/pre-ulcer care, vascular surveillance, recurrence prevention	Pre-ulcerative lesion, recurrent ulcer, lost pulse/waveform, footwear failure
